# Consumer species richness and nutrients interact in determining producer diversity

**DOI:** 10.1038/srep44869

**Published:** 2017-03-17

**Authors:** Sophie Groendahl, Patrick Fink

**Affiliations:** 1 University of Cologne, Cologne Biocenter, Workgroup Aquatic Chemical Ecology, Zuelpicher Strasse 47b, 50674 Cologne, Germany

## Abstract

While it is crucial to understand the factors that determine the biodiversity of primary producer communities, the relative importance of bottom-up and top-down control factors is still poorly understood. Using freshwater benthic algal communities in the laboratory as a model system, we find an unimodal relationship between nutrient availability and producer diversity, and that increasing number of consumer species increases producer diversity, but overall grazing decreases algal biodiversity. Interestingly, these two factors interact strongly in determining producer diversity, as an increase in nutrient supply diminishes the positive effect of consumer species richness on producer biodiversity. This novel and thus-far overlooked interaction of bottom-up and top-down control mechanisms of biodiversity may have a pronounced impact on ecosystem functioning and thus have repercussions for the fields of biodiversity conservation and restoration.

Biodiversity is frequently reported to strengthen ecosystem stability, ecosystem services and productivity^1^,^2^,^3^. An unprecedented worldwide decline in biodiversity, caused by anthropogenic factors, has been observed^4^,^5^. Despite their importance, our knowledge of the specific mechanisms that control biodiversity is limited^6^. Bottom-up regulation by nutrients is acknowledged to be one of the main factors which impact biodiversity and ecosystem functions^7^. Multiple studies have investigated the effect of nutrient enrichment on the biodiversity of primary producers^8^,^9^,^10^, but the question as to how biodiversity changes over a nutrient gradient, whether the relationship is unimodal or not, is still under hot debate^11^,^12^,^13^. Top-down regulation by consumers may also control biodiversity, in particular for primary producers^14^,^15^. However, most of the research on the effects of consumers on producer diversity has focused on grazing intensity rather than on consumer diversity^10^,^16^,^17^. As consumer species often vary in their feeding modes and selectivity, it is surprising that the potential effects of consumer diversity on prey diversity have been largely neglected. It is well known that a loss of a consumer species can radically change entire ecosystems^18^, but we can only guess what the consequences of losing multiple consumer species would be. Furthermore, it is acknowledged that the mechanisms that regulate biodiversity are manifold. Nevertheless, researchers tend to focus on single factors, disregarding potential interactions and feedback loops which may even accelerate the loss of biodiversity. There has been a long debate whether producer diversity is controlled by the presence of consumers (top-down regulation) or by the availability of resources (bottom-up regulation). Recent studies however indicate that rather than being exclusive, these two control mechanisms interact with each other^10^,^15^. In marine and freshwater systems, consumer presence and nutrient supply were both found to affect the diversity of primary producers^10^,^15^,^19^. Still no study has to our knowledge investigated the effect of nutrient enrichment and consumer species richness on the biodiversity of primary producers simultaneously, therefore their potential interactions have remained elusive. We believe that at low nutrient levels, an increase in consumer species richness could lead to a more balanced nutrient regeneration and thereby increase the biodiversity of the producers. At high nutrient levels, the impact of consumer species richness might not be strong enough to counteract competitive exclusion among primary producers. This could result in a strong interaction of both bottom-up (nutrient supply) and top-down (consumer species richness) control mechanisms on primary producer biodiversity.

We here test three specific hypotheses (Fig. 1): H1) The relation between nutrient availability and primary producer diversity, species richness and evenness should follow an unimodal relationship, frequently called the “Hump-Backed Model” (HBM^14^,^15^), as nutrients are insufficient to support a diverse primary producer community at low nutrient levels, whereas a few highly competitive species dominate at high nutrient levels. H2) The biodiversity of primary producers, species richness and evenness should increase linearly with consumer species richness. This could be explained by a diversification of resources through nutrient regeneration and/or reduced competition between producers^14^. H3) The positive effect of consumer species richness and nutrient regeneration on producer diversity, species richness and evenness should diminish with increasing amounts of nutrients. An increase in nutrients might increase the productivity of the algal community and thereby limit the consumers’ ability to reduce competitive exclusion among the algal species. We conducted three consecutive laboratory experiments in a model system consisting of freshwater benthic algal communities and herbivorous invertebrate grazers to test these three specific hypotheses. Our findings matched those theoretical considerations strikingly well.

## Results

### Nutrient effects on primary producers (H1)

**Figure 2:**
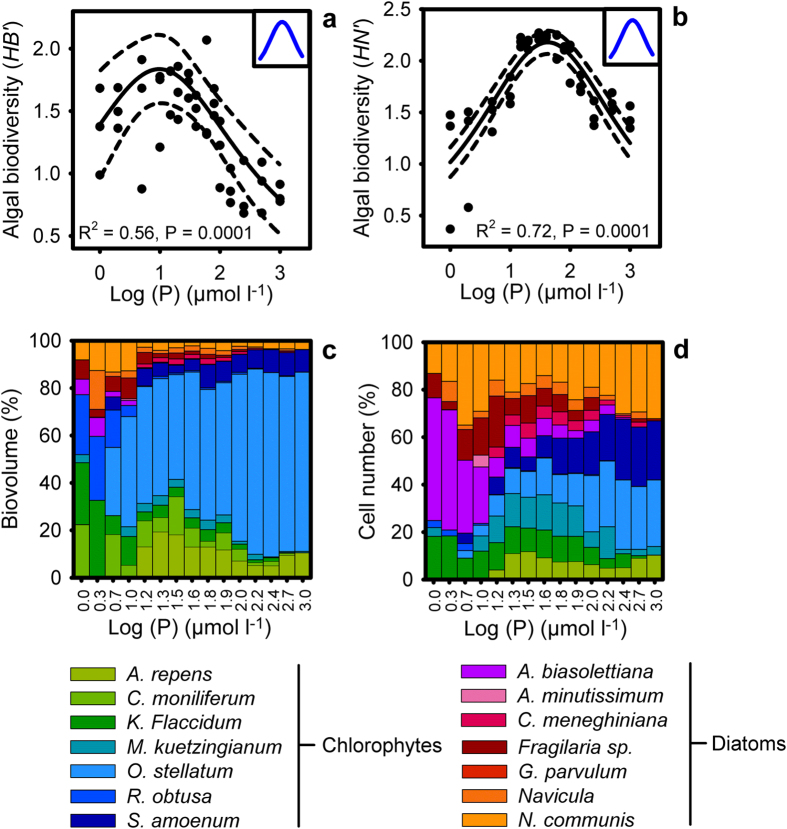
Impact of phosphorus availability on algal biodiversity and community composition. The Shannon diversity index based on algae biovolume *HB*′ (**a**) and cell number *HN*′ (**b**); the relative algal community composition based on biovolume (**c**) and cell number (**d**) was determined after three weeks in relation to a gradient of phosphorus (15 concentrations in triplicate). The results of the nonlinear regressions are represented as a solid line with 95% confidence intervals (dashed lines); the theoretical predictions are depicted in blue and are displayed in the insets in the upper right corners of panels a and b.

First, we investigated the relationship between nutrient (phosphorus, P) supply and algal biodiversity. The response variables were the Shannon diversity index based on algal biovolumes (*HB*′) and cell numbers (*HN*′) and Pielou’s index of evenness based on algal biovolumes (*JB*′) and cell numbers (*JN*′). All parameters followed the HBM as predicted by our first hypothesis (Fig. 2): We found that the dependence of algae biodiversity on P availability showed a highly significant unimodal relationship, in terms of *HB*′ (nonlinear regression, y = 1.69/(1 + ((x − 1.00)/1.74)^2^); Fig. 2a), *HN*′ (nonlinear regression, y = 2.18/(1 + ((x − 1.63)/1.52)^2^); Fig. 2b), *JB* (Supplementary Fig. S1a), *JN* (Supplementary Fig. S1b) and primary producer species richness (Supplementary Fig. S1c). Furthermore, an increase in P availability had pronounced effects on the algal community composition (Fig. 2c,d). Filamentous green algae tended to dominate the communities at higher P levels, whereas diatoms were more common at lower P levels (Fig. 2c,d). Moreover, the biovolume of the primary producers followed a sigmoidal pattern with increasing amounts of P and reached a plateau at approximately 40 μmol P l^−1^ (Supplementary Fig. S2), at which point light probably became the limiting factor for primary production.

### Consumer species richness effects on primary producers (H2)

**Figure 3:**
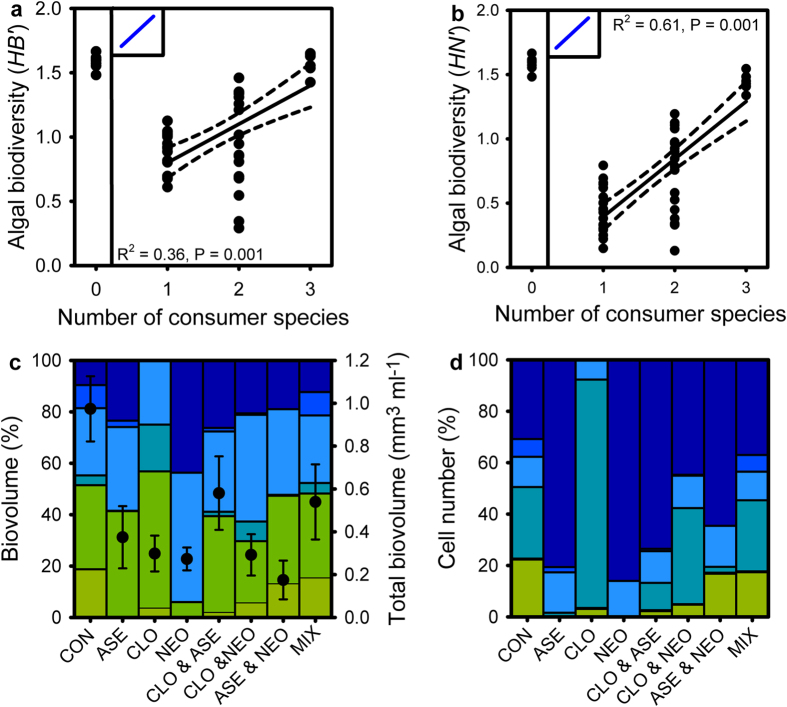
Impact of consumer species richness on algal biodiversity and community composition. Shannon diversity index based on algae biovolume *HB*′ (**a**, one consumer treatment N = 21; two consumer treatment N = 21; three consumer treatment N = 7) and cell number *HN*′ (**b**, one consumer treatment N = 21; two consumer treatment N = 21; three consumer treatment N = 7); the relative algal community composition (bars) based upon biovolumes (**c**) and cell numbers (**d**) and the absolute biovolume (**c**) (circles ± SD) (N = 6–8), for each consumer species (CON = control, ASE = *Asellus aquaticus*, CLO = *Cloeon dipterum*, NEO = *Neocaridina davidi*), was determined after three days. The results of the linear regression are represented as a solid line with 95% confidence intervals (dashed lines) in panels a,b. The consumer-free control treatment is depicted as 0 consumer species in panels a,b and is not included in the linear regression. The theoretical predictions are depicted in blue and displayed in the insets on the upper left corner of panels a,b.

Second, we investigated the effect of consumer species richness on the biodiversity of algal communities. The highest diversity was found in the control treatment. To separate the effect of grazer diversity from the overall effect of grazing pressure, the control treatment as excluded from the subsequent analyses We found a highly significant, positive linear relationship between consumer species richness and primary producer diversity, both in terms of *HB*′ (Fig. 3a) and for *HN*′ (Fig. 3b). Moreover, we found a significant positive relationship between consumer species richness and *JB*′ (Supplementary Fig. S3a), *JN*′ (Supplementary Fig. S3b) and primary producer species richness (Supplementary Fig. S3c, Table S2). The consumer species varied significantly in their preferences for specific algal taxa (Fig. 3c,d, Supplementary Fig. S4). In the one consumer species treatments we found that all consumer species significantly reduced the biovolume of *Aphanochaete repens, Oedogonium stellatum* and *Roya obtusa* (Supplementary Fig. S4). *Cloeon dipterum* did not decrease the biovolume of *Microthamnion kuetzingianum,* whereas the two other consumer species did (Supplementary Fig. S4). However, *C. dipterum* significantly reduced the biovolume of *Stigeoclonium amoenum* compared to the two other consumer species (Supplementary Fig. S4). *Neocaridina davidi* on the other hand preferred feeding upon *Closterium moniliferum* (Supplementary Fig. S4). The total algal biomass was significantly higher in the control treatment than in the three consumer species treatment (T-test, T_1,14_ = 4.38, P < 0.001, N = 8).

### Nutrients and consumer species simultaneous effects on primary producers (H3)

**Figure 4:**
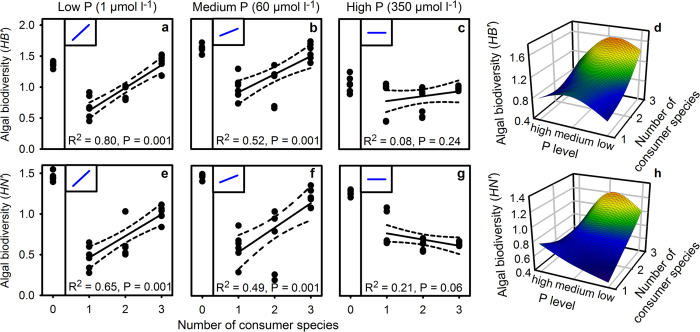
Impact of phosphorus concentration and consumer species richness on algal biodiversity. Shannon diversity index based on algae biovolume (*HB*′, **a**–**c**, N = 6) and cell numbers (*HN*′, **e**–**g**, N = 6) determined after one week in relation to low (**a,e**), medium (**b,f**) and high (**c,g**) initial phosphorus levels. Results of the linear regression are represented as a solid line with 95% confidence intervals (dashed lines). The consumer-free control treatment is depicted as 0 consumer species in panels a–c and e–g. The 3D plots (**d,h**) (N = 6) visualize the interactive effects of the number of consumer species and initial phosphorus levels on the mean Shannon diversity based on algal biovolumes *HB*′ (**d**) and cell numbers *HN*′ (**h**). The theoretical predictions are depicted in blue and are displayed in the insets in the upper left corners in the panels a–c and e–g.

In the final experiment, the two factors previously tested separately were combined in order to investigate whether or not interactions between bottom-up and top-down control mechanisms on primary producer diversity occur. The control treatment was not included in the following linear regressions. We found a highly significant interaction between nutrient enrichment and grazer species richness (Supplementary Table S1, Fig. 4).

**Figure 5:**
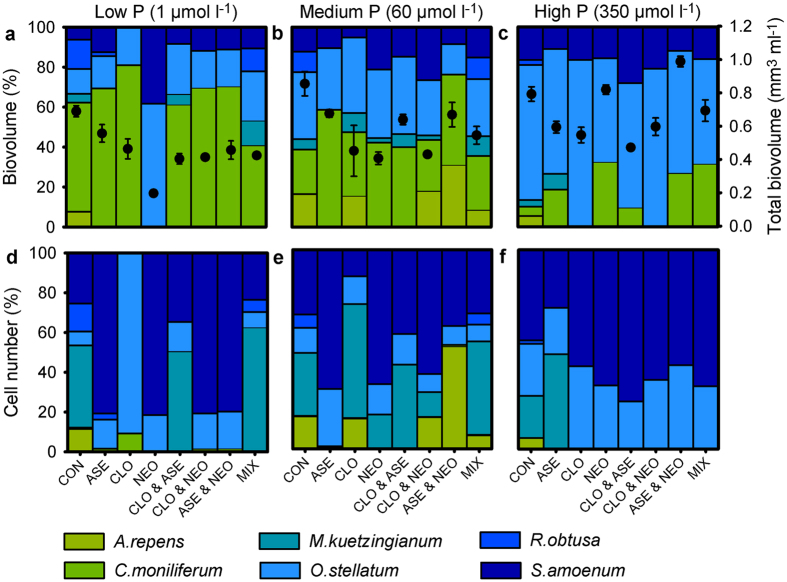
Impact of phosphorus concentration and consumer species richness on algal community composition. Graphs depict the relative algal community composition (bars) and the absolute biovolume (circles ± SD) (N = 2–6) after one week based upon algae biovolumes (**a–c**) and cell numbers (**d–f**) for each consumer species (CON = control, ASE = *A. aquaticus*, CLO = *C. dipterum*, NEO = *N. davidi*) at either low (**a,d**), medium (**b,e**) or high (**c,f**) initial phosphorus levels.

However, this pattern was only present at low (Fig. 4a,e) and intermediate (Fig. 4b,f) levels of dissolved P (Supplementary Table S1). Similarly, we found a significant positive relationship between consumer species richness and *JB*′, *JN*′ and primary producer species richness at low (Supplementary Fig. S5a,d,g) and medium phosphorus levels (Supplementary Fig. S5b,e,h), but not at high phosphorus levels (Supplementary Fig. S5c,f,i, Table S3). Again, *HB*′ and *HN*′ displayed a unimodal relationship with the P supply (Fig. 4d,h). The algal community structures were strongly affected by the P supply levels (Fig. 5), the filamentous green algae increased in dominance with phosphorus supply. Moreover, the algal biovolumes were significantly higher in the high P treatment than in the other treatments (two-way ANOVA, F_6,60_ = 27, P < 0.001, N = 6; Fig. 5).

## Discussion

As predicted by our hypothesis, our first laboratory experiment yielded an unimodal (humpbacked) relationship between nutrient availability and the biodiversity of the primary producer community. Evenness as well as species richness of the primary producer community showed almost identical patterns. Earlier experiments which attempted to link the diversity of primary producers to nutrient enrichment reported conflicting results: Biodiversity has been found to increase^8^ or decrease^20^,^21^ with nutrient loading. The most probable cause for these inconsistent findings of those previous studies is the rather narrow range of nutrient concentrations applied. This has previously been demonstrated in richness-productivity relationships^12^. Additionally, it may be inappropriate to apply the same nutrient gradient to different types of organisms (autotrophs and heterotrophs), as their capacity for physiological responses may vary strongly^21^,^22^. Previous studies demonstrated that N:P ratios may have pronounced effects upon primary producer diversity^23^. As we manipulated only the P supply in our experiment which caused a concomitant variation in the N:P supply ratio, we cannot entirely rule out that those changes in N:P also affected the primary producer diversity.

We hypothesized that primary producer diversity would increase linearly with consumer species richness. We found however that the presence of consumers decreased algal diversity in the second experiment. Still, an increase in consumer species richness sustained a higher level of biodiversity in the primary producers as the three consumer species treatment exhibited an algal biodiversity in the same range as the control treatment. In contrast to grazing pressure, which have been found to decrease primary producer species richness, but increase primary producer evenness in freshwater ecosystems^10^, consumer species richness did not induce different responses in the algae community in terms of evenness and primary producer species richness. Both evenness and species richness of the primary producer community decreased in the presence of grazers. However, an increasing number of consumer species sustained a higher primary producer evenness and species richness. Only a few studies have explicitly addressed the effects of consumer diversity on prey diversity^24^,^25^,^26^,^27^. Some of them observed either no effect or negative effects of consumer species richness on primary producer diversity^26^,^27^. These results might be explained by the similar feeding preferences of the consumer species utilized in those studies. Other studies (including ours) indicated an increase in the diversity of primary producers due to complementary feeding among the consumer species^24^,^25^. We found that complementary feeding resulted in a more even grazing pressure upon the algae species; the biodiversity of the algae community was thus conserved. Moreover, when the consumer species had an overlap in their feeding preferences, the consumption of the preferred algae (*A. repens* and *R. obtusa*) was decreased, possibly due to competition. This resulted in an increase in the biodiversity of the algal community. A more diverse consumer species community may also lead to a more balanced nutrient regeneration^28^ and hence to a feedback loop which could further promote the biodiversity of primary producers.

As predicted by our third hypothesis, eutrophication (i.e. nutrient addition) diminished the effects of increasing consumer species richness on primary producer diversity, evenness and species richness. Probably, the nutrient enrichment increased the productivity of the primary producer community and thereby limited the grazers’ ability to counteract the growth of more competitive algal species. Previous studies have demonstrated that an increase in grazing pressure reduces the biodiversity/species richness of primary producers, but this effect was weakened when nutrient availability was higher^10^,^15^,^29^ (but see ref. 30). The decline in biodiversity with increasing grazing pressure observed in previous experiments, may be caused by a higher likelihood of rare species being consumed^31^. This suggests that an increase in grazing pressure may have reduced the biodiversity in the low phosphorus treatment, were productivity was lower, but might have enhanced the biodiversity at high phosphorus levels.

We excluded the control treatment from the regression analysis in the second and third experiment as the duration of the experiments was too short for competitive exclusion to have a strong effect onto the algal communities in the absence of consumers. We believe that if the duration of the experiment would have been extended, the lack of disturbance in the control treatments would have resulted in a few competitive algae species dominating the community. Yet, we decided not prolong the experiments any further in order to avoid light from becoming the limiting factor for primary production. The control treatment was very much similar to the three consumer species treatment in terms of species richness and diversity. However, a reduction in consumption (e.g. due to territorial behaviour) in the three consumer species treatment cannot explain the similarities, since the total algal biovolume was significantly higher in the grazer-free controls than in the three consumer species treatment.

Obviously our experimental system with a mixture of pure cultures of benthic algae that were kept semi-suspended is somewhat artificial. We chose this strategy to avoid differential access to the dissolved nutrient pool for the different algal taxa which may have occurred in a structured substrate-attached biofilm. Nevertheless, our model system provided insights into the mechanisms which govern biodiversity that can be generalized to some degree to primary producer communities in general.

Our data strongly suggest that increasing nutrient levels lead to additive species losses by countervailing the potential for consumer species to sustain the biodiversity of primary producers. A decrease in producer diversity caused by eutrophication may result in reduced consumer diversity^32^, giving rise to a feedback loop of extinction across various trophic levels which may compromise the ability of these systems to retain their biodiversity. Thus, interactions between consumer species richness and resources have severe implications for ecosystems’ functioning and for conservation planning.

## Material and Methods

### Nutrient effects on primary producers (H1)

In the first laboratory experiment, 14 benthic algae species from the two dominant functional groups in biofilms (diatoms and chlorophytes) were selected from culture collections (Supplementary Table S2). Each strain was pre-cultured for five weeks in a climate chamber at 20 °C in WC medium^33^. The light-dark regime was set to 16:8 h at a light (PAR) intensity of 100 μmol photons m^−2^ s^−1^ (OSRAM L36W/830 and L36W/965), measured in air. The two other experiments were conducted under the same culture conditions. The experimental units consisted of Erlenmeyer flasks (300 ml), which were filled with 200 ml of modified sterile WC medium. Each flask received a different concentration of dissolved phosphate (K_2_HPO_4_ × 3H_2_O) as a source of phosphorus. Altogether, 15 levels of dissolved phosphorus (replicated three times along an exponential gradient) were added (Supplementary Table S3). KCl was added to compensate for osmotic differences between the experimental units after K_2_HPO_4_ addition (Supplementary Table S3). The flasks were inoculated with equal biovolumes of each of the 14 algal species at a total algal biomass of 3 × 10^6 ^μm^3^ ml^−1^. Conversion factors from the literature^34^,^35^ were used for biovolume estimates. A mean of 50–100 cell measurements of the respective species were taken to convert cell sizes into biovolumes for the different algal species (Supplementary Table S2). The Erlenmeyer flasks were gently shaken three times a week in order to achieve a homogeneous distribution of algae and nutrients within the flasks. The experiment was terminated after three weeks. Three 1 ml samples were taken from each unit, fixed with 150 μl of Lugol’s iodine solution and counted under an inverted microscope.

Shannon’s diversity index (*H*′) was calculated as (1):


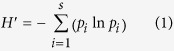


in which p_i_ is the proportion of individuals belonging to the i^th^ species^36^.

Pielou’s index of evenness was calculated as (2):


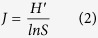


in which *H′* is the Shannon’s diversity index and S is the number of species^37^.

### Consumer species richness effects on primary producers (H2)

In the second laboratory experiment, six green algal species were used (Supplementary Table S2). The algae were pre-cultured in batch cultures for three weeks in WC medium. 150 ml of water were added to the units used in the experiments (Erlenmeyer flasks, 300 ml) together with 15.8 mm^3^ of each pre-cultured algal species. One to three species of invertebrate grazers (Supplementary Table S4) were added to the flasks. The guidelines for the use of animal behaviour for research and teaching (Animal Behaviour 83: 301–309) were followed in order to avoid any animal suffering. To ensure equal grazing pressure in all units, consumer species were added in a 1: 3: 4 ratio of *N. davidi: C. dipterum: A. aquaticus,* based upon their previously determined specific ingestion rates (unpublished data). A full factorial design was setup (Supplementary Table S5). The experiment was terminated after three days, to avoid total removal of biomass. Samples were taken and counted as described above. A few replicates could not be counted for technical reasons and thus were excluded from the diversity estimates.

### Nutrients and consumer species simultaneous effects on primary producers (H3)

In the third laboratory experiment, six green algal species (Supplementary Table S2) were pre-cultured in WC-medium for two weeks. 150 ml of modified WC-medium were added to the Erlenmeyer flasks (300 ml) used for the experiments. The concentration of dissolved phosphate was adjusted to three phosphorus levels (Supplementary Table S6). KCl was added to compensate for osmotic differences between the experimental units after K_2_HPO_4_ addition (Supplementary Table S6). 34.6 mm^3^ of each of the pre-cultured algal species were inoculated into the flasks. One to three species of invertebrate grazers (Supplementary Table S4) were added in the same ratios as in the previous experiment. To investigate the combined effect of nutrient and consumer species richness on algal biodiversity, a full-factorial design was set up (Supplementary Table S7). After one week, the experiment was terminated and samples were taken and counted as described above.

### Statistical Analysis

All regressions were conducted using SigmaPlot (v. 11, SYSTAT). The relationship between phosphorus availability and biodiversity of primary producers was tested via a nonlinear regression (Lorentzian 3-parameter curve). To test for the relationship between phosphorous availability and productivity (biovolume), a nonlinear regression was conducted (Sigmoid 3-parameter curve). Linear regressions were conducted to investigate the relationship between consumer species richness and primary producer diversity/primary producer species richness. One-way ANOVAs (factor grazer) using SPSS v. 23 (IBM, 2015) were conducted for each algal species, followed by a Tamhane T2 post-hoc test, which does not require homoscedasticity, to compare the specific feeding preferences between grazers. A t-test was performed using SigmaPlot in order to test for significant differences of the total algal biovolumes between the control treatment and the three consumer species treatment. To analyse the differences between algal biovolumes in the low, medium and high phosphorus treatments, a two-way ANOVA was conducted using SigmaPlot, followed by *post-hoc* comparisons with Tukey’s HSD. An ANCOVA was conducted in Statistica v. 10 to test for interactive effects between nutrient enrichment and consumer species richness on primary producer diversity, followed by *post-hoc* comparisons with Tukey’s HSD. The tests conducted were two-tailed and statistical significance was defined as P < 0.05. Prior to the statistical tests, all data were checked for homoscedasticity using Levene’s test.

## Additional Information

**How to cite this article**: Groendahl, S. and Fink, P. Consumer species richness and nutrients interact in determining producer diversity. *Sci. Rep.* **7**, 44869; doi: 10.1038/srep44869 (2017).

**Publisher's note:** Springer Nature remains neutral with regard to jurisdictional claims in published maps and institutional affiliations.

## Supplementary Material

Supplementary Information

Supplementary Dataset 1

## Figures and Tables

**Figure 1:**
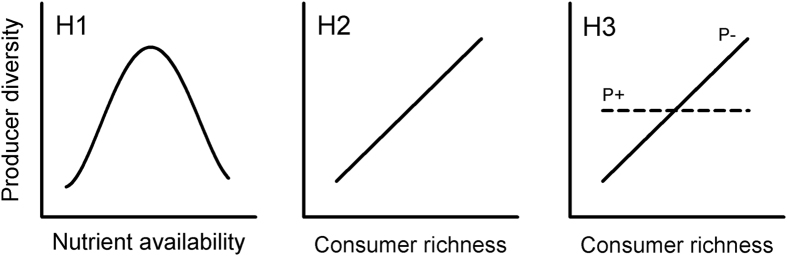
Conceptual diagram of the hypothesized effects of nutrients and consumer species richness on primary producer diversity. Producer diversity is highest at intermediate phosphorus levels (H1), modified from Worm *et al*.^15^; producer diversity has a positive correlation towards consumer species richness (H2), modified from Hillebrand and Shurin^14^; an increase in nutrient loading (P+) would weaken the positive correlation between producer diversity and consumer species richness (H3).
