# Supplementary material for: Consumer species richness and nutrients interact in determining producer diversity
Source: Sci Rep. 2017 Mar 17;7:44869. doi: 10.1038/srep44869 (PMC5356013; doi:10.1038/srep44869)
Supplement: Supplementary Information [file srep44869-s1.pdf]

Supplementary Information for

Consumer species richness and nutrients interact in determining producer diversity

Sophie Groendahl and Patrick Fink

correspondence to: sgroenda@uni-koeln.de

This file includes:

Supplementary Figures S1 to S5

Supplementary Tables S1 to S7

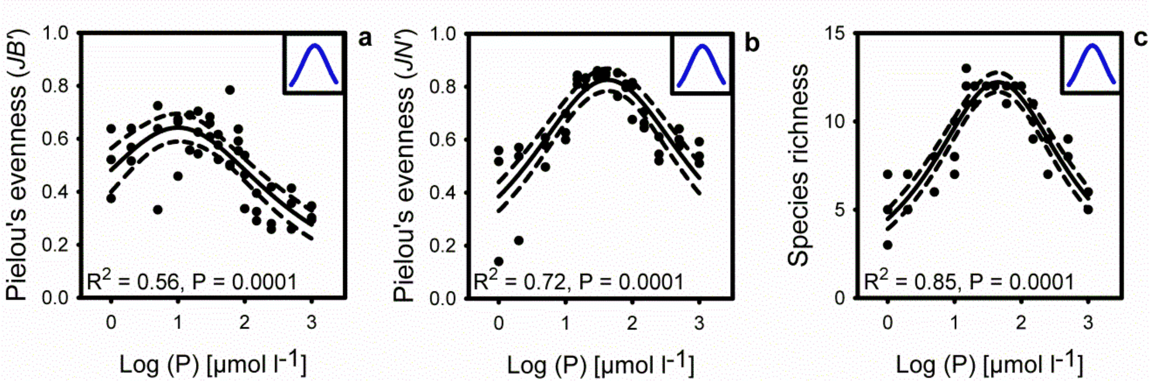

Supplementary Figure S1. Impact of phosphorus availability on algal evenness and species richness. Pielou's index of evenness based on algae biovolume *JB′* (nonlinear regression, $y = 0.64/(1+((x-1.00)/1.74)^2)$ (a), cell number *JN′* (nonlinear regression, $y = 0.83/(1+((x-1.63)/0.52)^2)$ (b) and the algal species richness (nonlinear regression, $y = 12.21/(1+((x-1.65)/1.525)^2)$ (c) were determined after three weeks in relation to a gradient of phosphorus (15 concentrations in triplicate). The results of the nonlinear regressions are represented as a solid line with 95 % confidence intervals (dashed lines); the theoretical predictions are depicted in blue and are displayed in the insets in the upper right corners.

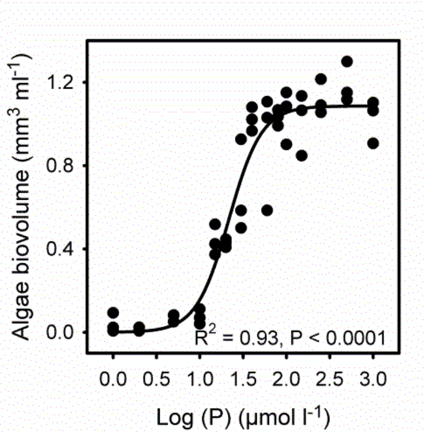

Supplementary Figure S2. Effects of phosphorus availability on total algal biovolume in the first experiment. Total algal biovolume determined after three weeks in relation to initial phosphorus concentrations (15 concentrations in triplicate) (sigmodial regression, y = 1.09(1+exp(-(x-1.34)/0.19))). Results of a nonlinear regression are represented as a solid line.

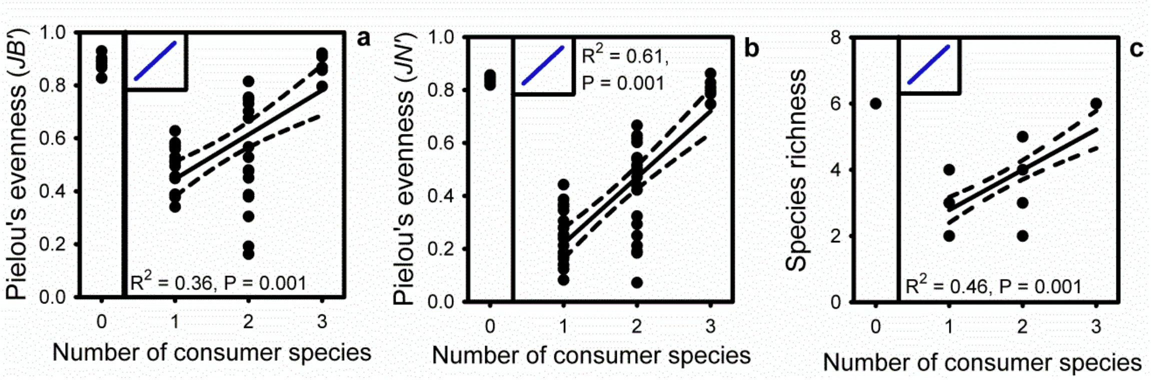

Supplementary Figure S3. Impact of consumer species richness on algal evenness and species richness. Pielou's index of evenness based on algae biovolume *JB′* (a) and cell number *JN′* (b) and the algal species richness (c) (one consumer treatment N = 21; two consumer treatment N = 21; three consumer treatment N = 7) for each consumer species (CON = control, ASE = *A. aquaticus*, CLO = *C. dipterum*, NEO = *N. davidi*), were determined after three days. The results of the linear regression are represented as a solid line with 95 % confidence intervals (dashed lines). The consumer-free control treatment is depicted as 0 consumer species. The theoretical predictions are depicted in blue and displayed in the insets on the upper left corner.

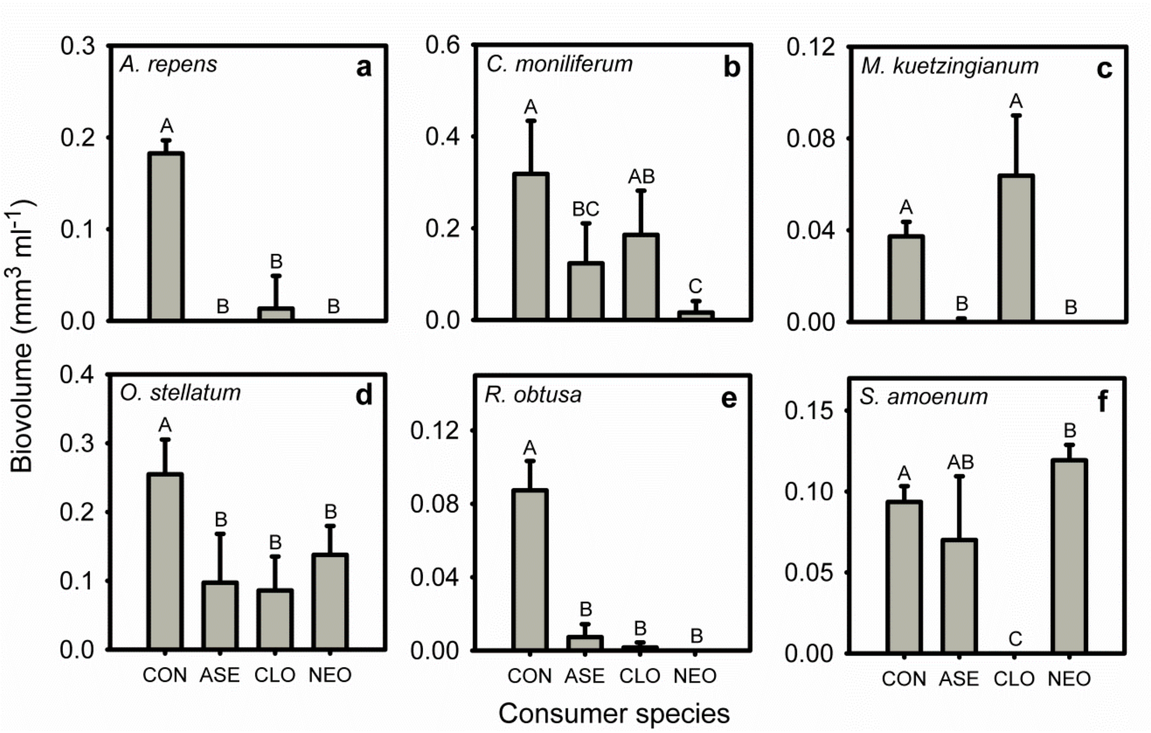

Supplementary Figure S4. Impact of grazing on single algal species in the second experiment. Bars represent algal biovolumes (mean ± SD) after three days for each consumer species (CON = control, ASE = *A. aquaticus*, CLO = *C. dipterum*, NEO = *N. davidi*) for *Aphanochaete repens* (a, F = 170, P < 0.001), *Closterium moniliferum* (b, F = 16, P < 0.001), *Microthamnion kuetzingianum* (c, F = 38, P < 0.001), *Oedogonium stellatum* (d, F = 16, P < 0.001), *Roya obtusa* (e, F = 167, P < 0.001) and *Stigeoclonium amoenum* (f, F = 53, P < 0.001). Means which were found to be significantly different after *post-hoc* comparisons (via the Tamhane T2 test) are labeled with different capital letters. N = 6 - 8.

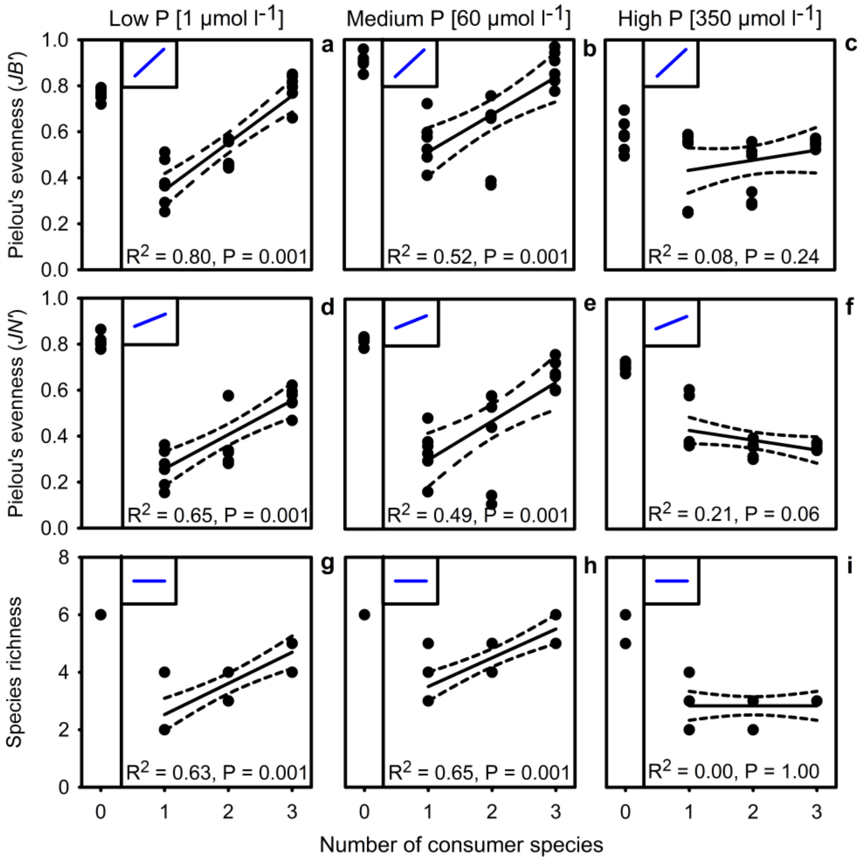

Supplementary Figure S5. Impact of phosphorus concentration and consumer species richness on algal evenness and species richness. Pielou's index of evenness based on algae biovolume (*JB'*, a - c) and cell numbers (*JN'*, d - f) and the algae species richness (g - i) (N = 6) determined after one week in relation to low (a, d, g), medium (b, e, h) and high (c, f, i) initial phosphorus levels. Results of the linear regression are represented as a solid line with 95 % confidence intervals (dashed lines). The consumer-free control treatment is depicted as 0 consumer species in panels. The theoretical predictions are depicted in blue and are displayed in the insets in the upper left corners.

Supplementary Table S1. ANCOVA on the interaction between number of consumer species and nutrient level on the biodiversity of algae (*HB'*). F-statistics and P-values are shown. d.f. = degrees of freedom. N = 6.

**Effect**	**df**	**F**	**P**
Intercept	1	40	0.0001
Nb of consumer species	1	41	0.0001
P-level	2	11	0.0001
Nb of consumer species x P-level	2	6	0.005

Supplementary Table S2. Origin and mean biovolume per cell of the algal species used in the experiments. The seven diatom and green algae species used in the first experiment, and the green algal species used (except for *K. flaccidum*) in the second and third experiments.

**Chlorophytes**	**Volume (μm^3)**	**Origin/ Strain**	**Diatoms**	**Volume (μm^3)**	**Origin/ Strain**
Aphanochaete repens	1760	CCAC/ M2227	*Achnanthes biasolettiana*	40	Lake Constance
Closterium moniliferum	*195050*	CCAC/ 2066 B	*Achnanthidium minutissimum*	60	Lake Constance
Klebsormidium flaccidum	460	CCAC/ 2007 B	*Cyclotella meneghiniana*	420	CCAC/ 0039
Microthamnion kuetzingianum	290	CCAC/ 0087 B	*Fragilaria sp.*	*250*	CCAC/ M 2678
Oedogonium stellatum	4660	CCAC/ 2231 B	*Gomphonema parvulum*	110	CCAC/ 2682 B
Roya obtusa	2730	CCAC/ 0219 B	*Navicula sp.*	490	CCAC/ 1772 B
Stigeoclonium amoenum	660	CCAC/ 3255 B	*Nitzschia communis*	200	CCAC/ 1762 B

Supplementary Table S3. Concentration of phosphate and potassium added in the first experiment. 15 different concentrations of phosphate were added to yield a nutrient gradient; in treatments with lower phosphate addition, higher amounts of potassium chloride were added in order to keep the medium's osmolarity at similar levels.

P [µmol l^{-1}]	K_2HPO_4 [mg l^{-1}]	KCl [mg l^{-1}]
1	0.2	149.0
2	0.5	148.8
5	1.1	148.4
10	2.3	147.6
15	3.4	146.9
20	4.6	146.1
30	6.8	144.6
40	9.1	143.1
60	13.7	140.2
80	18.3	137.2
100	22.8	134.2
150	34.2	126.7
250	57.1	111.8
500	114.1	74.6
1000	228.2	0.0

Supplementary Table S4. Origin and size range of consumer species in the second and third experiments.

Consumer species	Size (mm)	Origin
Asellus aquaticus (Linné, 1758)	5 - 9	Pond on campus of the University of Cologne, NRW, Germany
Cloeon dipterum (Linné, 1761)	5 - 8	Pond on campus of the University of Cologne, NRW, Germany
Neocaridina davidi (Bouvier, 1904)	9 - 15	Gillbach, in the outflow of RWE Power AG, Bergheim, NRW, Germany

Supplementary Table S5. Setup of second experiment. To ensure equal grazing pressure in all units, consumer species were added in a 1: 3: 4 ratio of *N. davidi: C. dipterum: A. aquaticus*

Treatment	Consumer species combinations	Replicates	No. of individuals
**Control**		8	none
**One consumer**	*A. aquaticus*	8	12
	C. dipterum	8	9
	N. davidi	8	3
**Two consumer species**	*A. aquaticus, C. dipterum*	8	6, 5
	A. aquaticus, N. davidi	8	6, 2
	C. dipterum, N. davidi	8	5, 2
**Three consumer species**	*A. aquaticus, C. dipterum, N. davidi*	8	4, 3, 1

Supplementary Table S6. Concentration of phosphorus and potassium added in the third experiment. Three different levels of phosphorus were used. Potassium chloride was added in order to keep the medium's osmolarity at similar levels.

P level	P [µmol l^{-1}]	K_2HPO_4 [mg l^{-1}]	KCl [mg l^{-1}]
low	1	0.2	149.0
medium	60	13.7	140.2
high	350	79.9	96.9

Supplementary Table S7. Setup of experiment three. P-levels of low (1 µmol P l^{-1}), medium (60 µmol P l^{-1}) and high (350 µmol P l^{-1}) availability. To ensure equal grazing pressure in all units, consumer species were added in a 1: 3: 4 ratio of *N. davidi: C. dipterum: A. aquaticus.*

Treatment	P-level	Consumer species combinations	Replicates	No. of individuals
Control	low		6	
One consumer species	low	*A. aquaticus*	2	12
	low	*C. dipterum*	2	9
	low	*N. davidi*	2	3
Two consumer species	low	*A. aquaticus, C. dipterum*	2	6, 5
	low	*A. aquaticus, N. davidi*	2	6, 2
	low	*C. dipterum, N. davidi*	2	5, 2
Three consumer species	low	*A. aquaticus, C. dipterum, N. davidi*	6	4, 3, 1
Control	medium		6	
One consumer	medium	*A. aquaticus*	2	12
	medium	*C. dipterum*	2	9
	medium	*N. davidi*	2	3
Two consumer species	medium	*A. aquaticus, C. dipterum*	2	6, 5
	medium	*A. aquaticus, N. davidi*	2	6, 2
	medium	*C. dipterum, N. davidi*	2	5, 2
Three consumer species	medium	*A. aquaticus, C. dipterum, N.davidi*	6	4, 3, 1
Control	high		6	
One consumer species	high	*A. aquaticus*	2	12
	high	*C. dipterum*	2	9
	high	*N. davidi*	2	3
Two consumer species	high	*A. aquaticus, C. dipterum*	2	6, 5
	high	*A. aquaticus, N. davidi*	2	6, 2
	high	*C. dipterum, N. davidi*	2	5, 2
Three consumer species	high	*A. aquaticus, C. dipterum, N. davidi*	6	4, 3, 1
